# Deubiquitinase USP35 stabilizes BRPF1 to activate mevalonate (MVA) metabolism during prostate tumorigenesis

**DOI:** 10.1038/s41420-022-01231-x

**Published:** 2022-11-10

**Authors:** Guowen Lin, Tianrun Huang, Xiaobo Zhang, Gangmin Wang

**Affiliations:** 1 Department of Urology, Fudan University Shanghai Cancer Center, Shanghai, China; 2 Department of Oncology, Shanghai Medical College, Fudan University, Shanghai, 200032 China; 3 Shanghai Municipal Hospital of Traditional Chinese Medicine, Shanghai University of Traditional Chinese Medicine, Shanghai, China; 4 Department of Emergency The People’s Hospital of Guangxi Zhuang Autonomous Region, Nanning, China; 5 Department of Urology, Huashan Hospital, Fudan University, Shanghai, China

**Keywords:** Prostate cancer, Ubiquitylation

## Abstract

The mutual interplay between epigenetic modifications and metabolic rewiring contributes to malignant features of prostate adenocarcinoma (PRAD). This study aimed to uncover the biological roles of deubiquitylase USP35 in PRAD and find effective epigenetic or metabolic targets. Bioinformatic tools or methods revealed that USP35 is upregulated in PRAD samples and correlates with inferior prognosis. The in vitro and in vivo assays suggested that USP35 could enhance malignant features of PRAD cells. Mechanistically, we found that USP35 could directly deubiquitinate and stabilize BRPF1 proteins. USP35 depends on accumulated BRPF1 proteins to accelerate cell growth, stem-like properties, and migration in vitro and in vivo. Interestingly, high BRPF1 could bind to promoter of SREBP2 and activate the SREBP2 transcriptional capacity. Therefore, USP35/BRPF1 aixs could promote expressions of mevalonate (MVA) metabolism signature in a SREBP2-dependent manner. USP35 depends on BRPF1 to maintain the activity of mevalonate metabolism in PRAD cells. Last of all, we observed that targeting BRPF1 or using MVA inhibitor (atorvastatin) are effective to suppress USP35^high^ PRAD in vivo tumor growth. USP35 is an indicator of MVA metabolic signature in PRAD. Collectively, our study highlighted the USP35/BRPF1/SREBP2 axis in modulating MVA metabolism in PRAD, suggesting the significance of BRPF1 or MVA as the potential therapeutic targets for PRAD treatment.

## Background

As the leading reason of cancer-associated death among males worldwide, prostate cancer remains to be the second most commonly diagnosed malignancy [1, 2]. According to the 2022 cancer statistics, the estimated new cases would come up to 268,490, along with cancer-related 34,500 deaths [3]. Although patients with localized tumors in early stages commonly obtain favorable prognosis, the 5-year progression-free survival in metastatic cases dramatically decreases to nealry 30% [4, 5]. The androgen-deprivation (ADT) therapy is regarded to be the standard treatment for advanced prostate cancer, but it has limited efficacy in castration-resistant prostate cancer (CRPC) [1, 6]. Meanwhile, limited treatment efficacy correlates tightly with the development of even more specific subtypes, like neuroendocrine prostate cancer (NEPC) [7]. As a result, how to identify effective targets to treat lethal prostate cancer is a highly desired issue to be elucidated.

Normal protein degradations and turnover contribute to the balance of cellular homeostasis and participate in various biological events [8]. As reported, lysosomal-mediated proteolysis and proteasome-mediated degradation are the two essential proteolytic manners which are highly conserved in eukaryotes [9]. The ubiquitin proteasome system (UPS) contributes to destructions of nearly 80% intracellular proteins, thus manipulating a series of biological events, like cell cycle, migration, metastasis, and drug resistance [10, 11]. Intensive studies have reported that aberrant UPS could lead to prostate cancer progression and aggressive features. Of note, the gene encoding the E3 ubiquitin ligase substrate-binding adaptor SPOP is frequently mutated in primary prostate cancer that leads to accumulations of multiple onco-proteins, including BRD4, AR, NANOG, Caprin1, or GLI [12, 13]. Besides, the E3 ubiquitin ligase STUB1 is down-regulated in prostate cancer, leading to activated JMJD1A/AR signaling that enhances cancer progression and enzalutamide resistance [14]. In contrast, a series of deubiquitinating enzymes (DUBs) exert functions in the processes during prostate tumorigenesis that have provided therapeutical targets for treatment [15]. For instance, USP16, known as a deubiquitinase, was strongly associated with the c-Myc gene signature to serve as a novel deubiquitinase of c-Myc, thereby enhancing the castration-resistant prostate cancer cell proliferation [16]. The deubiquitinase BAP1 is reported to physically bind to and deubiquitinate PTEN, playing an essential role in prostate cancer suppression [17]. Furthermore, USP33 is found to be overexpressed in prostate cancer cells that could inhibit the Lys48 (K48)-linked polyubiquitination of DUSP1, mediating the docetaxel resistance of CRPC [18]. Therefore, we speculated that whether there exist other DUB members that influence progression of prostate cancer.

In the current study, we found that USP35 is a novel oncogenic DUB in prostate tumorigenesis, which has never been reported. We reported that BRPF1 is a substrate of USP35 and USP35/BRPF1 axis promotes malignant features of prostate cancer via activating the MVA pathway. Our data collectively suggested the possible therapeutic implications of targeting USP35/BRPF1 as an innovative strategy to improve the overall prognosis in prostate cancer patients.

## Results

### USP35 expressed highly in PRAD samples that correlates with inferior prognosis

**Fig. 1:**
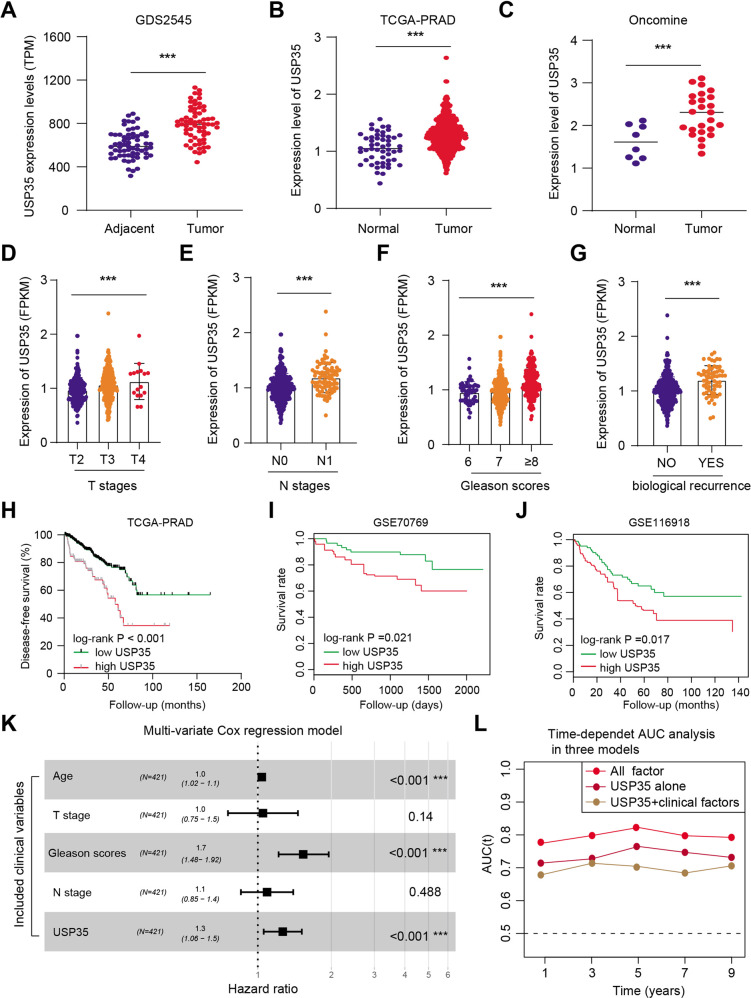
USP35 is aberrantly up-regulated in PRAD samples and correlates with worse prognosis. **A** The USP35 expression data between adjacent and tumor tissues derived from GDS2545 in GEO (Adjacent group: 63, Tumor group: 65) was compared and shown. **B** Differential analysis of USP35 levels derived from TCGA-PRAD cohort between normal and tumor samples were compared and shown. **C** The expression levels of USP35 in Oncomine were searched and compared. The correlation analysis between USP35 levels and clinical variables was assessed, including T stages (**D**), N stages (**E**), Gleason scores (**F**) and biological recurrence (**G**). **H**–**J** DFS, PFS, or OS are assessed based on high or low expression of USP35 levels and log-rank t test was utilized. **K** Multi-variate Cox regression model was conducted the assess the independent risk factors via integrating age, TN stages, Gleason scores and USP35 levels. **L** The time-dependent receiver operating characteristic curve (ROC) was conducted and generated to evaluate the predictive efficiency of USP35 in PRAD prognosis. * *p* < 0.05, ** *p* < 0.01, *** *p* < 0.001.

First of all, we queried the expression data of USP35 in GDS2545 derived from the Gene Expression Omnibus (GEO) database, observing that USP35 levels were higher in 65 tumor samples as compared to 63 adjacent normal tissues (Fig. 1A). The clinical information of PRAD samples was summarized in Table S1. In line with the findings, we also analyzed that USP35 expressed highly in PRAD samples in TCGA-PRAD and Oncomine datasets, respectively (Fig. 1B, C). Besides, we collected the information of clinical characteristics for the PRAD patients to conduct the correlation analysis. As expected, high USP35 expressions were observed in patients with advanced T or N stages (Fig. 1D, E). Also, patients with a definite biochemical recurrence or ≥8 Gleason scores showed elevated levels of USP35 (Fig. 1F, G). Moreover, Kaplan–Meier survival curves analysis revealed that patients with high USP35 levels bear worse disease-free survival (DFS) in TCGA-PRAD cohort (*p* < 0.001), worse overall survival (OS) in GSE70769 (*p* = 0.021), as well as worse progression-free survival (PFS) in GSE1169181 (*p* = 0.017) (Fig. 1H–J). Lastly, we also conducted the multi-variate Cox regression analysis by integrating several hazard clinical variables in TCGA-PRAD cohort. Compared with other variables, like age, TN stages, or Gleason scores, USP35 is an independent prognostic factor for predicting DFS of PRAD patients (Fig. 1K). The time-dependent receiver operating characteristic curve (ROC) further demonstrated that combination of USP35 and other clinical variables could reach higher predictive efficiency, as compared to USP35 or clinical variables respectively (Fig. 1L). In conclusion, our bioinformatic analysis suggested that USP35 is up-regulated in PRAD samples and possesses a tight correlation with a worse prognosis.

### USP35 enhances tumorigenesis and stemness potentiality of PRAD cells

**Fig. 2:**
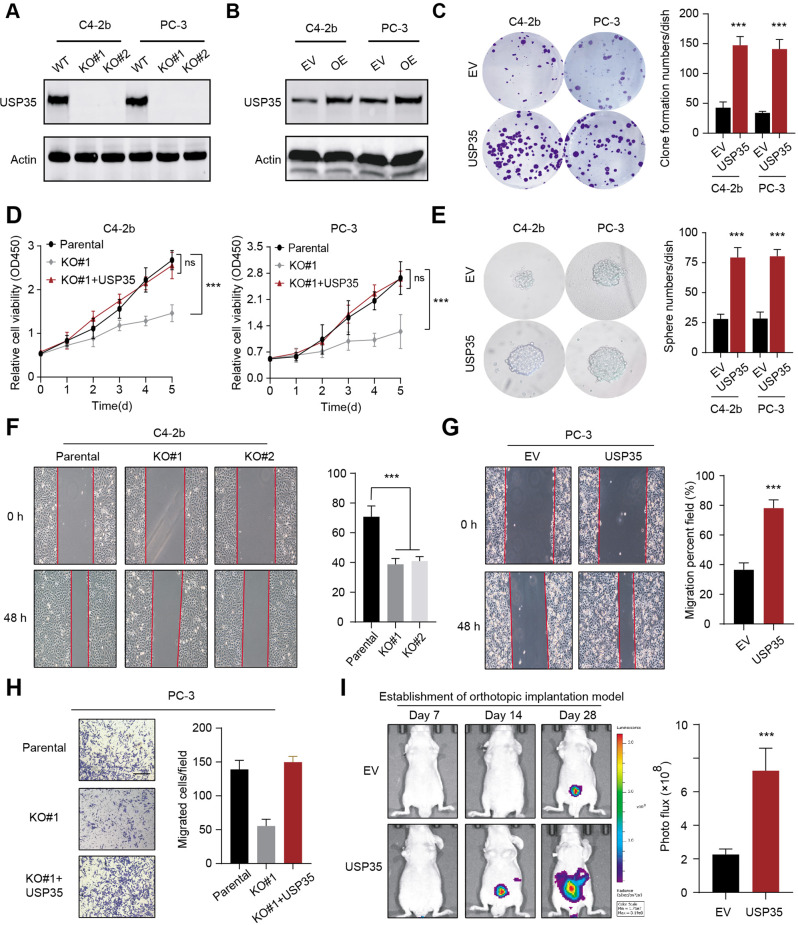
BRPF1 potentiates PRAD progression in vitro and in vivo. **A** BRPF1 was deleted in PRAD cell lines (C4-2b and PC-3) that are confirmed by western blotting. **B** Besides, BRPF1 overexpression was detected in PRAD cell lines (C4-2b and PC-3). **C** Proliferation abilities of C4-2b and PC-3 were enhanced via ectopic expression of USP35, which were shown by colony formation assay and statistical data. **D** In contrast, CCK-8 assays revealed that BRPF1 loss impeded cell growth, whereas BRPF1 overexpression rescued this effect. **E** The stem-like properties were also elevated via USP35 overexpression, and the statistical data was shown on the right. **F** The wound healing assay showed that BRPF1 loss impeded the migration rates of C4-2b cells in 0 h and 24 h, individually. The statistical data was shown on the right. (**G**) Conversely, BRPF1 overexpression enhanced the migration efficacy relative to cells transfected with vector. **H** Transwell assay revealed that USP35 inhibited the migration ability, while ectopic expression of USP35 rescued the impaired ability of PC-3 cells. **I** The growth of orthotopic prostate tumors was enhanced by USP35 overexpression and the in vivo BIL luciferase signals were detected and compared. The statistical data was shown on the right. * *p* < 0.05, ** *p* < 0.01, *** *p* < 0.001.

Based on the above bioinformatic findings, we intended to assess the USP35 functions in prostate cancer. First of all, we deleted USP35 in two prostate cancer cell lines C4-2b and PC-3 via sgRNA-mediated CRISPR/Cas9 KO technology (Fig. 2A). In contrast, we generated stable USP35-overexpressing cell lines via lentivirus infection (Fig. 2B). Next, we started to determine the roles of USP35 in PRAD proliferation. The colony formation assay exhibited that USP35 overexpression notably promoted the number and sizes of PRAD cell colonies in two cell lines (Fig. 2C). USP35 depletion could decrease PRAD cell proliferation rates relative to parental control cells that could be restored by USP35 overexpression, as suggested by the cell viability assays (Fig. 2D). Meanwhile, we also found that USP35 could further enhance stem cell-like properties in PRAD cells, as evidenced by the sphere formation assays (Fig. 2E). The wound-healing assay also indicated that USP35 deletion resulted in decreased migration rates of cells relative to parental control cells (Fig. 2F). Conversely, ectopic expression of USP35 robustly potentiated the migration efficacy of cells as compared to cells transfected with vector (Fig. 2G). Transwell assays also indicated that ectopic expression of USP35 in USP35-deficient cells could completely rescue the restricted migration abilities (Fig. 2H). Last of all, to further evaluate USP35 roles in prostate tumorigenesis, we generated an orthotopic prostate tumor model in which C4-2b cells were injected into the prostate gland of nude mice. The in vivo bioluminescence (BIL) signals were used to indicate the growth of orthotopic prostate tumors. In accordance to our speculations, USP35 overexpression could result in the formation of larger prostate tumors relative to those in control mice, suggesting that USP35 significantly promoted tumorigenesis (Fig. 2I). Collectively, these data indicated that USP35 promotes the proliferation, migration, and stemness properties of PRAD cells both in vitro and in vivo.

### USP35 interacts with and deubiquitinates BRPF1 in PRAD cells

**Fig. 3:**
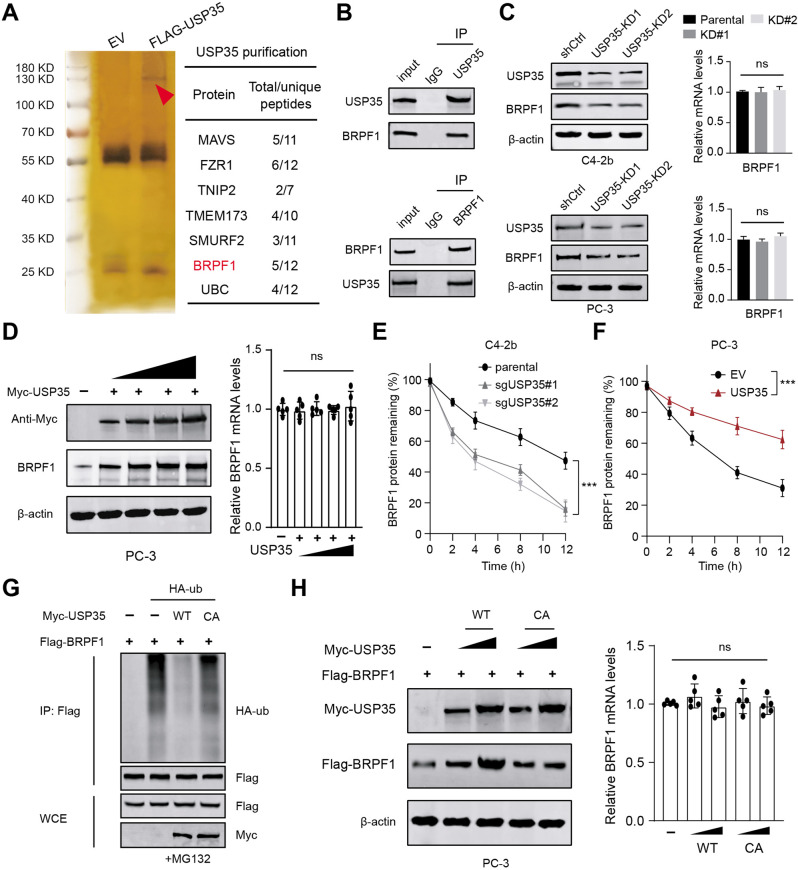
USP35 interacts with and deubiquitinates BRPF1. **A** Vector or FLAG-HA-USP35 plasmid was transfected in C4-2b cells for 24 h. After treatment of 10 μM MG132 for 4 h, cell lysate was immunoprecipitated with anti-FLAG and anti-HA beads, and the proteins interact with USP35 were screened. Silver staining picture was shown in the left panel and representative proteins were selected to show in the right panel. **B** The Co-immunoprecipitation (co-IP) assay was conducted the detect the endogenous interactions between USP35 and BRPF1 proteins in C4-2b. **C** Western blotting assay showed the decreased BRPF1 proteins in USP35-deleted C4-2b cells relative to parental control cells. The qPCR assay showed the mRNA levels of BRPF1 in three groups. **D** The C4-2b cells were transfected with increased amounts of Myc-USP35 plasmids and the protein levels of BRPF1 were detected by western blotting assay. The corresponding BRPF1 mRNA levels were also detected by RT-qPCR assays. **E** The parental and USP35-loss C4-2b cells were treated with 50 μg/ml cycloheximide (CHX) and harvested at different time points. At each time point, the intensity of BRPF1 was normalized to the intensity of actin and then to the value at 0 h. **F** In contrast, the CHX assay revealed that USP35 overexpression could prolong the half-time of BRPF1 proteins in cells transfected with USP35 plasmids relative to control cells. **G** The in vivo ubiquitination assay was conducted in cells transfected with Flag-BRPF1, Myc-USP35 (WT, or CA), or HA-ub. The cells were treated with 20 μM MG132 for 8 h, and western blotting showed the products. **H** The C4-2b cells were co-transfected with Flag-BRPF1 and Myc-USP35 (WT, or CA), respectively. The western blotting and RT-qPCR assays were used to detect the protein and mRNA levels of BRPF1 in the indicated groups. * *p* < 0.05, ** *p* < 0.01, *** *p* < 0.001.

To clarify the downstream targets that exert the oncogenic functions of USP35 in PRAD, we generated the stable C4-2b cells with double-tagged FLAG-HA-USP35. Next, the USP35-containing protein complex was purified and isolated via Tandem Affinity Purification (TAP) method. We thus identified a list of peptides in the complex, including FZR1, TNIP2, MAVS, or BRPF1 (Fig. 3A). Considering that epigenetic deregulation contributes to PRAD progression and BRPF1 is less reported in PRAD, we thus decided to confirm the associations between USP35 and BRPF1. First of all, the co-immunoprecipitation (co-IP) analysis with an anti-USP35 indicated that USP35 could directly interact with BRPF1, implicating the endogenous interactions (Fig. 3B). Besides, we also detected that the BRPF1 proteins were decreased in USP35-deleted C4-2b cells relative to parental control cells (Fig. 3C). However, USP35 loss could not induce alterations of BRPF1 mRNA levels (Fig. 3C). Next, we observed an increased levels of BRPF1 proteins when C42-B cells were transfected with elevated doses of Myc-USP35 plasmids, suggesting that USP35 promotes BRPF1 levels in a dose-dependent manner (Fig. 3D). Intriguingly, high USP35 did not alter BRPF1 mRNA levels (Fig. 3D). Consistently, the cycloheximide (CHX) assays also indicated that USP35 deletion resulted in shorter half-life of BRPF1 proteins relative to those in parental control C4–2B cells (Fig. 3E). In contrast, USP35 overexpression significantly prolonged the half-life of BRPF1 proteins in C4-2B cells (Fig. 3F). As shown in Fig. 3G, only wild-type USP35, but not the C450A mutant, could sufficiently catalyze deubiquitination of BRPF1. Accordingly, only wild-type USP35, but not the C450A mutant, could promote the accumulations of BRPF1 proteins, not altering the corresponding mRNA levels of BRPF1 (Fig. 3G). Taken together, these data indicated that USP35 can function as a deubiquitinase for BRPF1.

### USP35 depends on BRPF1 to exert oncogenic roles in prostate tumorigenesis

**Fig. 4:**
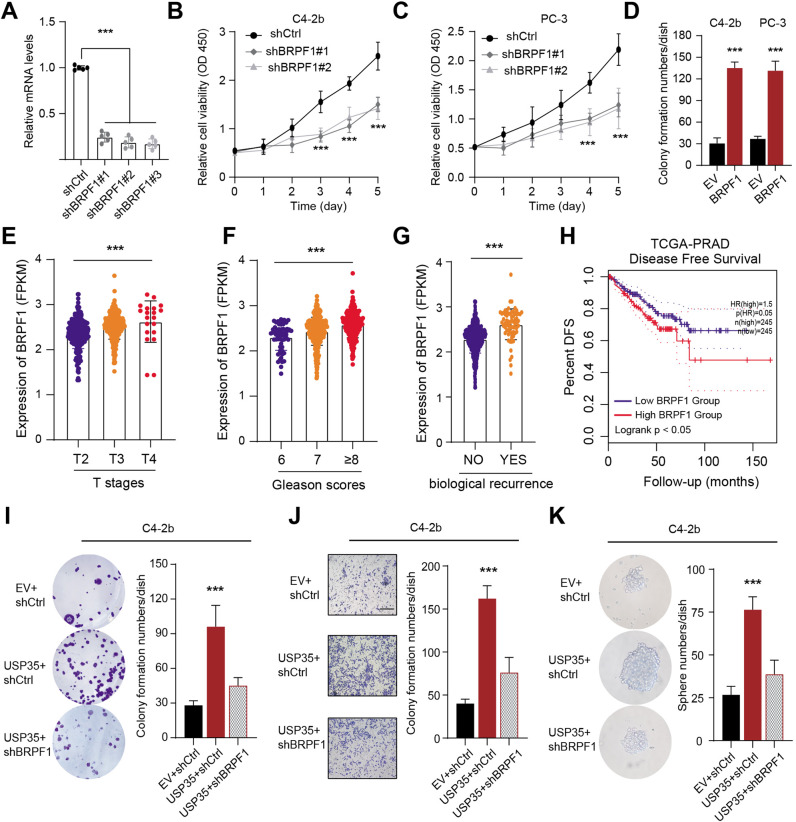
USP35 depends on BRPF1 to paly oncogenic roles in prostate tumorigenesis. **A** The RT-qPCR assays were conducted to detect BRPF1 mRNA levels in cells infected with specific shRNAs and shCtrl. **B**, **C** BRPF1 depletion significantly suppressed the growth of C4-2b and PC-3 cells, as revealed by CCK-8 assays respectively. **D** Quantification of colony formation assays in C4-2b and PC-3 cells transfected with BRPF1 and EV. **E**–**G** Correlation analysis was conducted to reveal the relationships between BRPF1 and T stages, Gleason scores, and biological recurrence in TCGA-PRAD samples. **H** Kaplan–Meier survival curve analysis was conducted to compare the DFS difference between BRPF1-high and BRPF1-low patients. **I**–**K** Colony formation assay, Transwell assay and sphere formation assay were conducted in three groups, including EV + shCtrl, USP35 + shCtrl, and USP35 + shBRPF1. * *p* < 0.05, ** *p* < 0.01, *** *p* < 0.001.

To further determine the biological roles in PRAD, we knocked down BRPF1 in C4-2b and PC-3 cells through lentiviral transduction method using three different BRPF1 short hairpin RNAs (Fig. 4A). The MTT assay showed that BRPF1 knockdown remarkably inhibited prostate cancer cell growth (Fig. 4B, C). However, BRPF1 overexpression could notably enhance cell colony formation ability of PRAD cells (Fig. 4D). In addition, bioinformatic analysis in TCGA-PRAD samples also suggested that high BRPF1 correlated highly with hazard clinical factors, like advanced T stages, high Gleason scores, as well as biological recurrence (Fig. 4E–G). Kaplan-Meier analysis suggested that patients with high BPRF1 levels had shorter DFS months as compared to those with low BRPF1 levels, as indicated by the log-rank test (Fig. 4H). Given that we have already generated USP35-overexpressing PRAD cell lines, we further knocked down BRPF1 in these cells. In line with the previous findings, USP35 is required for the proliferation, migration, and stem cell-like properties of PRAD cells, which could be largely abolished by BRPF1 KD (Fig. 4I–K). Collectively, these data implicated that USP35 depends on BRPF1 to enhance PRAD malignant progression.

### USP35/BRPF1 axis modulates mevalonate (MVA) metabolism via epigenetically inducing SREBP2 levels

**Fig. 5:**
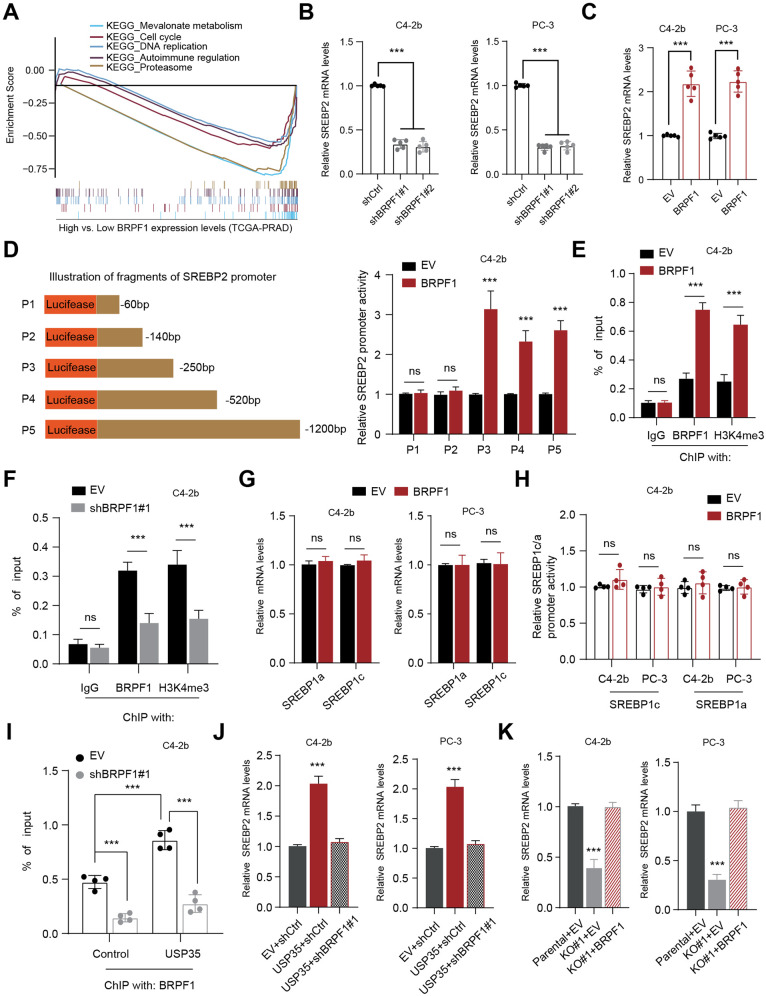
USP35/BRPF1 axis sustains mevalonate (MVA) metabolism via epigenetically activating SREBP2 expressions. **A** The TCGA-PRAD samples were divided into BRPF1-high and BRPF1-low groups according to the median data. GSEA analysis was conducted to find the enriched pathways related to BRPF1. **B** The RT-qPCR assays were conducted to assess the SREBP2 mRNA levels in C4-2b and PC-3 cells transfected with shBRPF1 and shCtrl, individually. **C** The RT-qPCR assays were conducted to assess the SREBP2 mRNA levels in C4-2b and PC-3 cells transfected with BRPF1 and EV. **D** The luciferase reporter gene assay was used to assess the activities of corresponding fragments of SREBP2 promoter in C4-2b cells, respectively. Cells were transiently transfected with control vector and BRPF1 vector (Mean±SD, *n* = 3). **E** ChIP assay was conducted to confirm the interaction of BRPF1 and the promoter region (P3) of SREBP2 in BRPF1-OE and control C4-2b cells, where IgG was the negative control. **F** ChIP assay was conducted to confirm the interaction of BRPF1 and the promoter region (P3) of SREBP2 in BRPF1-deficient and control C4-2b cells, where IgG was the negative control. **G** The RT-qPCR assays were used to assess the SREBP1a/c mRNA levels in C4-2b and PC-3 cells transfected with BRPF1 and EV, respectively. **H** Dual-luciferase assays were performed to detect the luciferase activity of the SREBP1a/c promoter in C4-2b cells (mean ± SD, *n* = 3). **I** C42-B cells were transfected with USP35 and EV plasmids for 12 h. The ChIP assay was performed to evaluate the interaction of BRPF1 with the promoter region of SREBP2 in two conditions. **J** The RT-qPCR assays were used to detect SREBP2 mRNA levels in three indicated groups: EV + shCtrl, USP35 + shCtrl and USP35 + shBRPF1#1. **K** The RT-qPCR assays were conducted to detect SREBP2 mRNA levels in three indicated groups: USP35-parental+EV, USP35-KO#1+EV, and USP35-KO#1 + BRPF1. * *p* < 0.05, ** *p* < 0.01, *** *p* < 0.001.

Given that BRPF1 is an epigenetic regulator that could induce specific genes expressions, we thus conducted the bioinformatic analysis based on sequencing expression data derived from TCGA-PRAD samples. Gene Set Enrichment Analysis (GSEA) implicated the tight interplay between BRPF1 and mevalonate (MVA) crosstalk in PRAD (Fig. 5A). Given that SREBP2 is the master regulator of MVA crosstalk, we thus intended to figure out the relationships between BRPF1 and SREBP2. Interestingly, BRPF1 KD could significantly reduce SREBP2 mRNA levels in PRAD cells (Fig. 5B). Besides, BRPF1 overexpression could promote SREBP2 mRNA levels (Fig. 5C). Meanwhile, we cloned a list of fragments of the SREBP2 promoter, and BRPF1 increased the luciferase activities of promoter fragments of P3, P4, and P5, but not the P1 or P2 (Fig. 5D). Thus, the region, ranging from −250 to −140 in the SREBP2 promoter, is the essential sequence binded and regulated by BRPF1. We also conducted the ChIP-qPCR assay to confirm that BRPF1 could directly bind to the SREBP2 promoter region to sustain the transcriptional activity, as implicated by the active H3K4me3 modification markers (Fig. 5E). Conversely, BRPF1 KD reduced the transcriptional activity at the SREBP2 promoter, as indicated by decreased H3K4me3 enrichment (Fig. 5F). To exclude the possibilities that BRPF1 could regulate other MVA transcriptional factors (TFs) like SREBP2, we detected that BRPF1 could not induce SREBP1a/c levels in PRAD cells (Fig. 5G). Along with these findings, we found that BRPF1 could not alter SREBP1a/c transcriptional activities (Fig. 5H). Given that USP35 could stabilize BRPF1, we thus wondered whether USP35 could rely on BRPF1 to modulate SREBP2 expressions. Apparently, we found that USP35 overexpression could enhance BRPF1 binding enrichment on the SREBP2 promoter region, which were notably suppressed with BRPF1 KD (Fig. 5I). In line with the results, we found that USP35 could enhance SREBP2 levels, and the increase could be completely abolished by BRPF1 KD (Fig. 5J). Conversely, USP35 deletion could suppress SREBP2 mRNA levels, which could be completely restored by BRPF1 overexpression in C4-2b and PC-3 cells (Fig. 5K). Taken together, these data implicated that USP35/BRPF1 axis could modulate MVA crosstalk in PRAD in a SREBP2-dependent manner.

### Targeting BRPF1-induced MVA crosstalk endows a therapeutical vulnerability for USP35^high^ PRAD

**Fig. 6:**
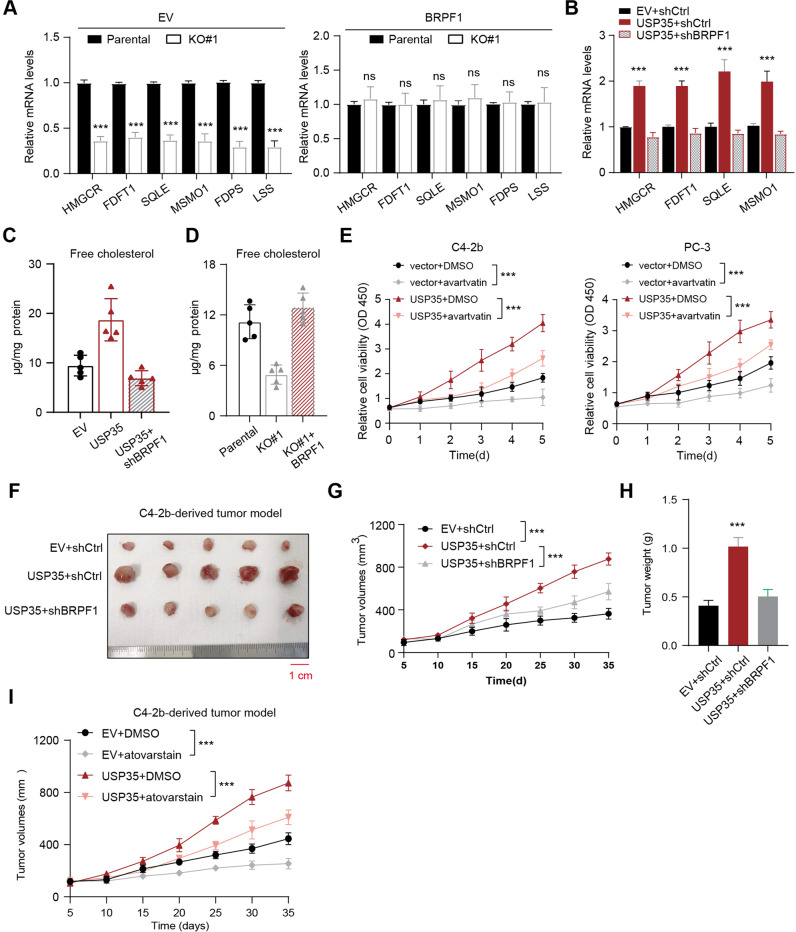
Targeting BRPF1/SREBP2/MVA axis endows a therapeutical vulnerability for USP35^high^ PRAD. **A** The RT-qPCR assays were performed to detect the MVA-signature genes in parental and USP35-KO cells. Besides, the USP35 loss cells were transfected with BRPF1 to further detect the mRNA levels of MVA genes. **B** The mRNA levels of MVA downstream genes (HMGCR, FDFT1, SQLE and MSMO1) were detected in three groups, including EV + shCtrl, USP35 + shCtrl and USP35 + shBRPF1#1. **C** The free cholesterol levels in cells were elevated by USP35, which could be notably suppressed by BRPF1 KD. **D** The free cholesterol levels in cells were suppressed by USP35-KO, which could be notably restored by BRPF1 overexpression. **E** The CCK-8 assays were used to detect the effect of avartvatin on cell growth, and assess the inhibitory effect of avartvatin on growth of USP35^high^ C4-2b or PC-3 cells. **F** Knockdown of BRPF1 inhibited USP35-induced subcutaneous tumor growth in nude mice derived from C4-2b cells. The representative tumor image was shown. **G** Quantification of tumor volumes in three indicated groups was exhibited and compared at the timepoints. **H** The tumor weight of mice derived from three groups was shown and compared. **I** The C4-2b-derived tumor model was generated to assess the efficacy of atovarstain on PRAD tumors, where the tumor volumes curve was shown. * *p* < 0.05, ** *p* < 0.01, *** *p* < 0.001.

Given that SREBP2 regulates a series of enzymes that sustain MVA metabolism activities, we observed that USP35 depletion could down-regulate the mRNA levels of SREBP2 downstream targets, named as MVA signature, including HMGCR, FDFT1, SQLE, MSMO1, FDPS and LSS (Fig. 6A). However, BRPF1 overexpression could completely restore the expressions of MVA signature (Fig. 6A). Meanwhile, USP35 could activate MVA signature and this effect could be completely abolished by BRPF1 KD (Fig. 6B). Consistently, we detected that the free cholesterol content in USP35-OE cells, was about 50% higher than that in controls, and the increase could be completely abolished upon BRPF1 KD (Fig. 6C). In contrast, USP35 loss suppressed the free cholesterol content and the decrease could be largely restored with BRPF1 overexpression (Fig. 6D). In addition, we utilized the atorvastatin to inhibit the MVA pathway and found that atorvastatin alone could restrict the growth of PRAD cells relative DMSO treatment (Fig. 6E). Meanwhile, in line with our biological findings, atorvastatin could further largely suppress the growth of USP35-OE PRAD cells (Fig. 6E). Given that we have found the USP35/BRPF1/SREBP2 axis in regulating MVA pathway during prostate tumorigenesis, we thus intended to further test the translational significance for treating PRAD. We generated the C4-2b-derived tumor model and found that targeting BRPF1 could significantly suppress USP35^high^ in vivo PRAD growth, as shown by tumor volumes and weight (Fig. 6F–H). Lastly, we also found that atorvastatin could also suppress the in vivo growth of tumors derived from USP35^high^ PRAD cells, as compared to those derived from control cells (Fig. 6I). Collectively, these results implicated that suppressing BRPF1-induced MVA crosstalk provides a therapeutical vulnerability for USP35^high^ PRAD.

**Fig. 7:**
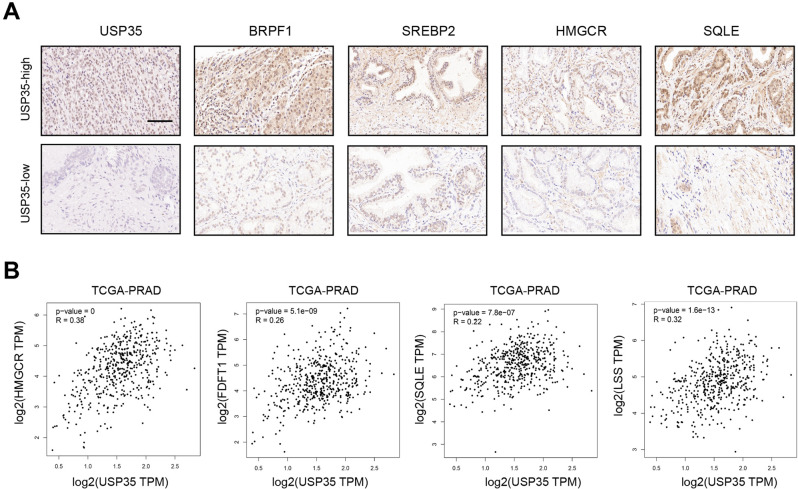
The clinical significance of the USP35/BRPF1 axis in activating tumor MVA signature in human PRAD. **A** The representative IHC images showed that USP35 levels were significantly associated with the expression of BRPF1 and SREBP2 in human PRAD specimens. **B** The associations among USP35 and MVA metabolic signature were further supported in TCGA-PRAD samples.

**Fig. 8:**
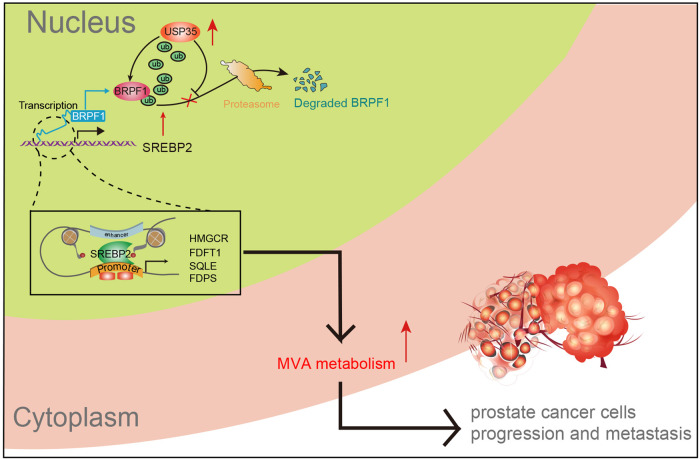
The graphical abstract showing the USP35/BRPF1/MVA crosstalk during prostate tumorigenesis.

Last of all, we collected some PRAD samples from our center and divided the samples into USP35^high^ and USP35^low^ groups. As shown in Fig. 7A, the positive associations among USP35, BRPF1, and SREBP2 were demonstrated via IHC method. We also assessed the positive relationships between USP35 and MVA signature in TCGA-PRAD samples (Fig. 7B). These data suggested that USP35 is an indicator for clinical PRAD samples with high MVA metabolism activity. We further illustrated the USP35/BRPF1/MVA axis in prostate cancer cells in Fig. 8.

## Discussion

Recently, USP35 is emerging as a hotspot target in cancer research. Zheng Tang et al. found that USP35 is abundant in human lung cancer tissues and cell lines that modulates iron homeostasis and ferroptosis through maintaining the stability of ferroportin (FPN) proteins [19]. Besides, deubiquitinase USP35 could restrain STING-mediated interferon signaling in ovarian cancer, highlighting the potential associations between USP35 and CD8^+^ T cell infiltrations [20]. Moreover, AKT-activated USP35 enhanced ERα stability by interacting and deubiquitinating ERα, suggesting that USP35 may be a therapeutical vulnerability for ER^+^ breast cancer with endocrine resistance [21]. Considering USP35 correlates tightly with tumor progression, whether USP35 could modulate other oncogenic features, like metabolic remodeling, stem-like properties is still unknown. Besides, the roles of USP35 in PRAD have never been defined. In the current study, we utilized the bioinformatic algorithms to uncover that USP35 is also up-regulated in PRAD samples and high USP35 levels correlated with hazard clinical characteristics, including TN stages, Gleason scores, or biological replase. Patients with high USP35 have poor prognosis. We further demonstrated that USP35 enhanced cancer cell growth, migration ability and stem-like properties in vitro and in vivo. Mechanistically, USP35 interacts with and stabilizes BRPF1 proteins, relying on accumulated BRPF1 to exert its oncogenic functions. Functional enrichment analysis proposed the tight interplay between BPRF1 and MVA signaling in PRAD samples. BPRF1 could bind to promoter of SREBP2 to activate the transcriptional activity. Thus, USP35/BRPF1 axis modulated the MVA crosstalk via induction of SREBP2. Last of all, in vitro and in vivo models suggested that suppressing BRPF1-mediated MVA crosstalk proved to be effective to inhibit USP35^high^ PRAD growth.

As reported, epigenetic modifications induce heritable alterations in gene expressions without changes of DNA sequences, including DNA methylation, histone modifications, as well as microRNAs (miRNA) [22]. Previous studies have identifed a series of epigenetic regulators that impact PRAD progression or drug resistance [23, 24]. Huairui Yuan et al. reported that SETD2 methylates EZH2 which promotes EZH2 degradation and SETD2 deficiency promotes a Polycomb-repressive chromatin state that renders cells to obtain metastatic potentiality in prostate cancer [25]. Besides, SPOP-mutant prostate cancers often accumulate BET proteins, including BRD2, BRD3 and BRD4 [26]. Elevated BRD4 could mediate chromatin remodeling effect to amplify androgen receptor (AR) downstream signaling and promote progression of CRPC or BET inhibitor resistance [27]. Recently, researchers also found that bromodomain-containing protein BRD9, one subunit of SWI/SNF complex, could interact with AR and CCCTC-binding factor (CTCF) to control AR-dependent gene expressions, highlighting the translational significance of nontoxic BRD9 inhibitors in PRAD treatment [28]. The bromodomain and PHD finger containing 1 (BRPF1) belongs to an epigenetic reader, and the bromodomain of BRPF1 may contribute to the chromatin binding and target specificity of the MOZ/MORF complex. Previous studies have indicated that BRPF1 is essential for embryonic development. Besides, BRPF1 loss also decreased the expressions of multipotency genes, like Slamf1, Mecom, Hoxa9, Hlf, or Gfi1, suggesting that BRPF1 is essential for the development of fetal hematopoietic stem cells [29]. Additionally, defective mutations of BRPF1 lead to intellectual disability and facial dysmorphisms in humans [30]. However, the associations between BRPF1 and PRAD progression is unknown. In this study, we conducted the bioinformatic analysis to find that BRPF1 is an oncogenic factor that correlated with PRAD progression. High BRPF1 contributes to tumor growth and malignant progression of PRAD, which is in accordance with findings in other solid tumors, like hepatocellular carcinoma (HCC) [31]. In addition, we uncovered the epigenetic regulations between BRPF1 and SREBP2, in which BRPF1 could epigenetically elevate the transcriptional activity of SREBP2. BRPF1 could directly activate the MVA pathway that provides a novel role of BRPF1 in tumor lipid metabolism.

As the essential hallmark of solid tumors, elevated cholesterol and lipid synthesis are considered to play important roles in metabolic rewiring during cancerous transformation [32]. As a precursor for bile acid and steroid hormone biosynthesis, cholesterol participated in constructions and function of cell membranes. Of note, the cholesterol-related metabolites play regulatory roles as signaling molecules in tumor progression. As well known, the mevalonate (MVA) metabolism employs acetyl-CoA to produce sterols and isoprenoids that are essential to potentiate tumor progression [33]. The sterol regulatory elementbinding protein (SREBP) family of transcription factors control the MVA pathway [34]. Mechanistically, SREBP2 is cleaved and translocated, induced by cholesterol depletion, to the nucleus to activate the transcription of MVA signature. As reported, the lipogenesis regulator SREBP2 directly induces transcription of the iron carrier Transferrin (TF), reducing reactive oxygen species (ROS), and lipid peroxidation, thereby resulting in resistance to inducers of ferroptosis in melanoma [35]. Besides, ZMYND8 and SREBP2 drive the enhancer-promoter interaction to facilitate the recruitment of Mediator complex, thus upregulating MVA pathway genes and enhancing colon cancer progression [36]. Intriguingly, in line with our results, Donge Tang et al also confirmed that KDM6A drives prostate tumorigenesis via activating SREBP1c-mediated lipid metabolism [37]. In this study, we highlighted the SREBP2-dependent MVA crosstalk is activated by USP35/BRFP1 axis to drive tumor progression. The in vitro and in vivo data suggested that targeting MVA is effective to suppress PRAD progression and further inhibited the USP35^high^ tumor growth. Previous studies have reported that the MVA pathway inhibitors (statins) exert beneficial efficacy in some specific colon cancer patients [36]. Accordingly, we speculated that targeting MVA (statins) would a valuable strategy to ameliorate USP35^high^ prostate cancer progression.

We still have some concerns in the current study that need to be further explored. First of all, owing to limited financial foundations, we did not thoroughly assess the inhibitory efficacy of atorvastatin in more pre-clinical models. The patient-derived tumor xenograft (PDX) models with high or low USP35 were needed to assess the efficacy of atorvastatin in patients with distinct genetic backgrounds. Besides, whether USP35 regulates other biological aspects of prostate cancer remains unclear, like immune cells infiltrations or bone metastasis. In addition, the inhibitors for BRPF1 are now available, including GSK-5959, or IACS-9571 [38]. We intended to assess the clinical efficacy of BRPF1 inhibitors in PRAD models in the following researches. Last of all, we are still uncertain about the relationships between USP35/BRPF1/MVA axis and castration resistance in prostate cancer. We would design other assays in the future to confirm whether USP35-mediated MVA metabolism could influence the progression of Castration-Resistant Prostate Cancer (CRPC). We need to collect more PRAD samples to validate the translational significance of USP35/BRPF1/SREBP2 axis in prognosis classification.

## Conclusion

Taken together, our study revealed the biological roles and prognostic significance of USP35 in PRAD. USP35 deubiquitinates BRPF1 to activate SREBP2 expressions, thereby strengthening the MVA crosstalk in PRAD progression. Thorough understanding of USP35/BRPF1/SREBP2 axis provides valuable strategies for PRAD treatment and prognosis classification.

## Methods and materials

### Cell culture

We obtained the 293T, C4-2b, 22Rv1, and PC-3 cells from the American Type Culture Collection (ATCC). The 293T cells were maintained in DMEM with 10%(v/v) fetal bovine serum (FBS), while C4-2b and PC-3 cells were maintained in RPMI 1640 with 10%(v/v) FBS.

### Tissue specimens

The 100 clinical prostate cancer specimens from patients were obtained from the department of urology, Huashan hospital (Shanghai, China). All patients underwent radical prostatectomy resection between January 2015 and September 2020. Patients who were previously diagnosed with other cancers and who received neoadjuvant treatments before surgery were not included. Histopathological diagnoses were made according to the WHO criteria. Written informed consent was obtained from each patient in this study for use of their tissues prior to the acquisition of the specimens. Ethical approval was granted by the Research Ethics Committee of Huashan hospital.

### Stable cell line construction and cell culture

pX459 plasmid was used to clone guide oligos targeting USP35 gene. C4-2b cells were plated and transfected with pX459 constructs for 24 h. After transfection for one day, 1 μg/ml puromycin was used to screen cells for 3 days. Living cells were seeded in 96 well plate by limited dilution to isolate monoclonal cell line. The knock out cell clones are screened by Western blot. Sequences of specific sgRNAs are listed as the following: sgUSP35#1: F: 5′-CACCGCACACGACTCGCAGTAGTAG-3′, R: 5′-AAACCTACTACTGCGAGTCGTGTGC-3′. sgUSP35#1: F: 5′-CACCGCTACTACTGCTATGCCCGTG-3′, R: 5′-AAACCACGGGCATAGCAGTAGTAGC-3′. For the knockdown assay, BRPF1 shRNAs were purchased from Sigma-Aldrich (MO, USA). shRNA plasmids were co-transfected with packaging constructs according to the manufacturer’s instruction to package the lentivirus. C4-2b and PC-3 cells were incubated with lentivirus for 72 h. The KD efficiency was confirmed by qRT-PCR assay. The shRNA sequences targeting BRPF1 were listed as the following: shBRPF1#1: CCGCATCAGCATCTTTGACAA; shBRPF1#2: CGCTACTTGAACTTTGATGAT; shBRPF1#3: CGTACTTTGAGAGTCACAATA.

### CCK-8 and colony formation assay

Cell proliferation was performed using the CCK8 kit following the manufacturer’s instruction (Jiangsu KeyGENBioTECH Corp., Ltd, China). Three thousand to five thousand prostate cancer cells were seeded into each well of a 96-well plate. Ten microliter CCK8 reagent was added to each well at the indicated time and incubated at 37 °C for 2 h. The absorbance at 450 nm was recorded with a 96-well plate reader. For the colony formation assay, thousand prostate cancer cells were seeded into each well of a 6-well plate and incubated at 37 °C for 10–14 days. The cells were then fixed, stained with 0.2% crystal violet, and imaged. Clones that consisted of at least 50 cells were considered as one colony.

### Real-time reverse transcription PCR (qRT-PCR)

Total RNA was isolated from cells using the TRIzol reagent (Tiangen), and cDNA was reversed-transcribed using the Superscript RT kit (TOYOBO) following the manufacturer’s instructions. PCR amplification was performed using the SYBR Green PCR master mix Kit (TOYOBO). All quantitations were normalized to the level of endogenous control GAPDH.

### Wound healing assay

The 4 × 10^4^ C4-2b or PC-3 cells were seeded in each well of a 6-well plate, and cells were scratched with a 1 ml pipette tip after confluent. After washed with PBS slightly, the images were captured by using a microscope equipped with a digital camera. The images were recorded again using the same microscope after 24 h.

### In vivo ubiquitination assay

For the in vivo ubiquitination assay, HEK293T cells were transiently co-transfected with indicated plasmids. After 24 h, cells were lysed with 100 μl lysis buffer (2% SDS, 150 mM NaCl and 10 mM Tris-HCl, pH 8.0), boiled for 20 min. 900 μl dilution buffer (150 mM NaCl, 1% Triton, 2 mM EDTA and 10 mM Tris-HCl, pH 8.0) was added. The samples were incubated with M2 or HA beads at 4 °C for 90–120 min with rotation. Then the beads were boiled after extensive washing with washing buffer (1 M NaCl, 1% NP40, 1 mM EDTA and 10 mM Tris-HCl, pH 8.0), and resolved via SDS-PAGE gel for immunoblotting analysis.

### Western blotting assay

The indicated cultured cells were lysed with RIPA lysis buffer (Beyotime, Shanghai, China) supplemented with 1% of the mixture of protease inhibitor (Sigma, MO, USA). Proteins were isolated and transferred to PVDF membranes (Millipore) in equivalent amounts using 10% SDSPAGE. As loading controls, antibodies against β-actin (60004-1-Ig, Proteintech) were used. Antibodies used were: USP35 (Proteintech, 24559-1-AP); BRPF1 (Abcam, ab259840); SREBP2 (Abcam, ab30682); FLAG (Abcam, ab205606); Myc (Abcam, ab32072); HA (Abcam, ab9110).

### Reporter assay

Prostate cancer cells were co-transfected with SREBP2 firefly luciferase reporter plasmid, Renilla luciferase plasmid, BRPF1 and USP35 expression plasmids by polyethylenimine (PEI) according to the manufacturer’s instruction. After 24 h, cells were lysed and centrifugated, and luciferase activity was measured using the dual-luciferase reporter assay system (Promega, Madison, USA). Relative SREBP2 activity was calculated as firefly luminescence relative to Renilla.

### In vivo xenograft experiment

All animal experiments were conducted according to the NIH Guide for the Care and Use of Laboratory Animals. All BALB/c nude male mice (4–6 weeks of age) were obtained. All experimental procedures using animals were approved by the Institutional Animal Care and Use Committee of Fudan University. The indicated C4-2b cells (1 × 10^6^) were mixed with matrigel (1:1) and injected subcutaneously into the flanks of BALB/c nude male mice. Tumors were measured using calipers every 5 days and tumor volumes were calculated using length × width × width × 0.52. Tumor tissues were paraffin embedded and H/E stained. For the construction of orthotopic mice model, male Balb/c athymic nude mice were acclimated for 3 weeks before experimental manipulation. Pca cells (C4-2b) were grafted into the mouse left or right DP under a stereoscopic microscope shortly after the cell resuspension in Matrigel. A 1.5 cm transverse incision was made in the lower midline with microscissors above the presumed location of the bladder. Approximately 100 μL intraperitoneal liquid spilled and was sponged with cotton balls. The intestine was pushed upward into the abdominal cavity using a sterile cotton swab. Starting 2 weeks post-injection, tumor growth was monitored by ultrasound (US) every 5 days. Meanwhile, the BIL signals within Pca tumors could be also detected to monitor the growth of orthotopic tumors.

### Statistical analyses

For cell proliferation, cell colony formation, and cell migration, data were analyzed by unpaired Student’s t test. Kaplan–Meier plot was used for patients with log-rank test. Two-tailed unpaired Student’s t tests or Mann–Whitney U tests were applied for comparisons between two groups. The differences with * *p* < 0.05 or ** *p* < 0.01 were considered statistically significant. Statistical analyses were performed with Prism 8.0 (GraphPad Software) and R studio (Version 3.5.3).

## Supplementary information

Table S1

Original Data File

## Data Availability

The data used to support the findings of this study are available from the corresponding author upon request. The TCGA-PRAD data was obtained from the TCGA platform via GDC portal (https://portal.gdc.cancer.gov/).
