# Supplementary figures and images for: Deubiquitinase USP35 stabilizes BRPF1 to activate mevalonate (MVA) metabolism during prostate tumorigenesis
Source: Cell Death Discov. 2022 Nov 10;8:453. doi: 10.1038/s41420-022-01231-x (PMC9649703; doi:10.1038/s41420-022-01231-x)

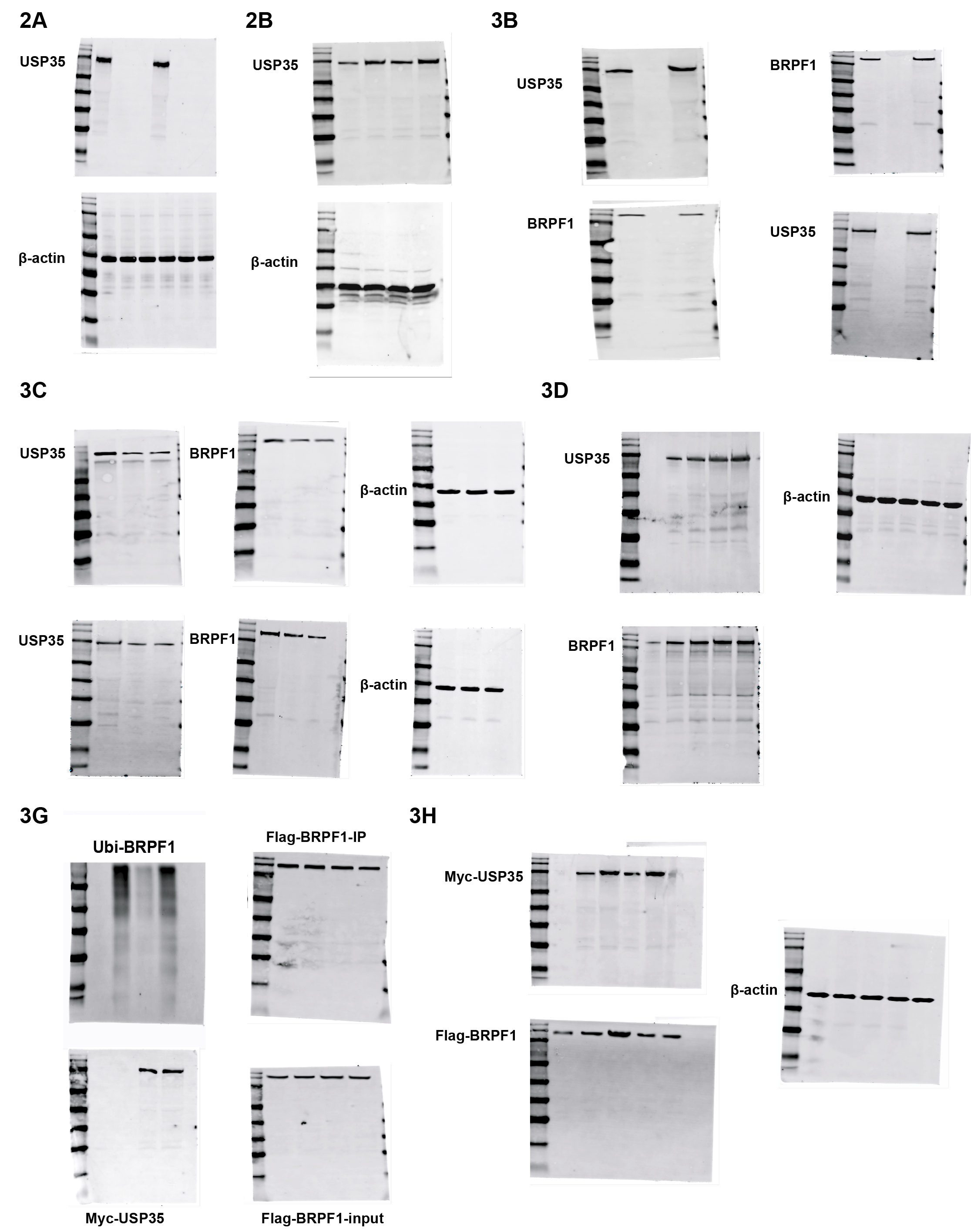

Supplement: Supplementary file 2 — Original Data File [file 41420_2022_1231_MOESM2_ESM.jpg]
